# Women 1.5 Times More Likely to Leave STEM Pipeline after Calculus Compared to Men: Lack of Mathematical Confidence a Potential Culprit

**DOI:** 10.1371/journal.pone.0157447

**Published:** 2016-07-13

**Authors:** Jessica Ellis, Bailey K. Fosdick, Chris Rasmussen

**Affiliations:** 1 Department of Mathematics, Colorado State University, Fort Collins, Colorado, United States of America; 2 Department of Statistics, Colorado State University, Fort Collins, Colorado, United States of America; 3 Department of Mathematics and Statistics, San Diego State University, San Diego, California, United States of America; Kyoto University, JAPAN

## Abstract

The substantial gender gap in the science, technology, engineering, and mathematics (STEM) workforce can be traced back to the underrepresentation of women at various milestones in the career pathway. Calculus is a necessary step in this pathway and has been shown to often dissuade people from pursuing STEM fields. We examine the characteristics of students who begin college interested in STEM and either persist or switch out of the calculus sequence after taking Calculus I, and hence either continue to pursue a STEM major or are dissuaded from STEM disciplines. The data come from a unique, national survey focused on mainstream college calculus. Our analyses show that, while controlling for academic preparedness, career intentions, and instruction, the odds of a woman being dissuaded from continuing in calculus is 1.5 times greater than that for a man. Furthermore, women report they do not understand the course material well enough to continue significantly more often than men. When comparing women and men with above-average mathematical abilities and preparedness, we find women start and end the term with significantly lower mathematical confidence than men. This suggests a lack of mathematical confidence, rather than a lack of mathematically ability, may be responsible for the high departure rate of women. While it would be ideal to increase interest and participation of women in STEM at all stages of their careers, our findings indicate that if women persisted in STEM at the same rate as men starting in Calculus I, the number of women entering the STEM workforce would increase by 75%.

## Introduction

Across the world there is tremendous need for more workers with degrees in science, technology, engineering, or mathematics (STEM). The U.S. President’s Council of Advisors on Science and Technology (PCAST) report predicts over the next decade approximately one million more STEM graduates above and beyond the current graduation level will be needed in order to meet the demands of the U.S. workplace [1]. The report also argues that simply increasing the retention of STEM majors by 10% would make considerable progress towards meeting this need. Similar projections have been made in the United Kingdom [2].

In the United States and elsewhere, first-year college and university mathematics courses often function as a bottleneck, preventing large numbers of students from pursuing a STEM career [3–5]. Introductory math courses, such as Calculus I, have repeatedly been linked to students’ decisions to leave STEM majors [6–8]. While calculus is not the only hurdle faced by potential U.S. STEM graduates, it is both one of the most challenging obstacles and a necessary first step on the way to a STEM career.

There has been a growing body of work investigating student persistence in STEM [3–9]. A common perception is that students leave STEM majors because of poor academic ability and that calculus functions as a course that “weeds out” mathematically incapable students [6, 10]. However, research suggests that switching from a STEM major to a non-STEM major is not an event, but a process based on a collection of curricular, instructional, and cultural issues [11, 12]. Seymour and her colleagues identified a number of these issues, including conceptual difficulties, poor instruction, inadequate preparation, and language barriers [11, 13]. More recent work suggests that a combination of student level variables (such as youth STEM-interest, student demographics, identity, socioeconomic status, and secondary school preparation), instructor level variables (such as pedagogy and teacher-student relationships), and institution level variables (such as student support once in college) together contribute to a student’s decision to persist in STEM or not [9, 12, 14–18]. A student’s experience in introductory courses is only one of a number of factors related to the decision to persist in a STEM field, and calculus is only one of a number of these introductory STEM courses. However, better understanding of how calculus instruction impacts STEM persistence and how different populations are differentially affected is an important step in increasing the STEM workforce.

**Fig 1:**
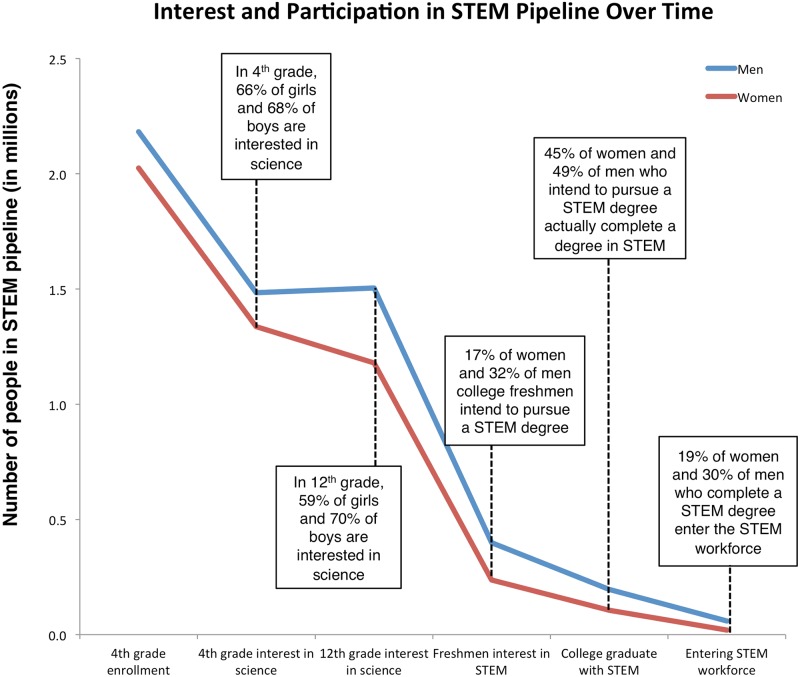
Participation of women in STEM.

In addition to this established “leaking STEM pipeline”, a number of populations exit the pipeline at higher rates than others and are thus underrepresented in STEM across all career stages. Women are an especially interesting under-represented group to investigate because they represent roughly 50% of the general population but only 25% of the overall STEM workforce. This percentage varies across specific STEM disciplines, with women holding 40% of the physical and life science jobs in 2009, about one-quarter of the computer science and math jobs, and only 14% of the jobs in engineering [19]. Although fourth-grade boys and girls report similar rates of interest in science, by twelfth-grade 59% of women and 70% of men report such an interest [20]. By the time students enter college, 17% of women intend to study a STEM field compared to 32% of men [21]. An estimated 40–60% of students who begin a STEM degree actually complete one, and of those only 35% are completed by women [21, 22]. Combined, these decreases in women’s participation in STEM lead to women making up only 25% of STEM workforce [23] (see Fig 1 and Table 1 for the derivation of this figure). In looking specifically within academia, these patterns persist, and although more women are entering academic positions than before, women continue to be an underrepresented minority in many STEM fields [30, 31]. Studies indicate that while there exists no bias against women in hiring for tenure track positions [32], women are not afforded the same opportunities, such as elite postdoctoral positions, that men are that help them be attractive for top academic positions [33].

**Table 1:** STEM participation by gender from primary school to the STEM workforce.

	Year	Male	Female	Source
4th grade enrollment	1999	2,182,000	2,025,000	[24]
4th grade interest in science %	2005	68%	66%	[25]
4th grade interest in science #		1,483,760	1,336,500	
12th grade enrollment	2007	2,149,000	1,998,000	[26]
12th grade interest in science %	2008	70%	59%	[20]
12th grade interest in science #		1,504,300	1,178,820	
Undergraduate freshmen enrollment	2009	2,132,000	2,457,000	[27]
4-year college enrollment %	2009	58%	56%	[28]
4-year college enrollment #		1,236,560	1,375,920	
4-year freshmen interest in science %	2009	32%	17%	[21]
4-year freshmen interest in science #		399,409	236,658	
Bachelor’s degrees in STEM	2012	196,763	106,005	[22]
STEM workforce		59,040	19,680	[23, 29]

As the U.S. faces a STEM graduate deficit, it is critical we understand why women and men are not completing STEM degrees at comparable rates and why both genders are not persisting with STEM degrees. In this study, we examine the role of Calculus I in STEM persistence for all students, focusing specifically on the gender gap. If a student elects not to take Calculus II, he or she is effectively choosing to exit the STEM pipeline. In the U.S. Calculus II is required for most STEM disciplines. Although there are some STEM degree programs that do not require Calculus II and some non-STEM degree programs that do require Calculus II, intentions to continue studying calculus after Calculus I can serve as a rough proxy for continuing to study STEM.

## Methods

The data used for this study comes from a unique, large-scale and in-depth national survey of Calculus I conducted under the auspices of the Mathematical Association of America. Colleges and universities were selected to participate using a stratified random sample of two- and four-year undergraduate colleges and universities during the 2010 Fall term. The San Diego State University Institutional Review Board (IRB) approved the study. The protocol number is 496064. Participant responses were de-identified prior to analysis.

Preparation of the surveys included a literature review leading to a taxonomy of potential dependent and independent variables followed by constructing, pilot testing and refining the survey instruments [34]. Administration of the surveys were restricted to what is known as “mainstream” calculus, the calculus course designed to prepare students for studying engineering or the physical sciences. Until now, there has been very little large-scale data collected on who elects to study Calculus I or on the effect of this course on student persistence in STEM.

Students were surveyed at the beginning and end of the Calculus I term and asked if they intended to take Calculus II. One year later, students were asked if they had taken or enrolled in Calculus II. Based on students’ responses, we identified students who initially intended to take Calculus II and noted whether this intent was maintained or not after Calculus I (see S1 Table for more information). Those who maintained their initial intention to take more calculus are referred to as Persisters and those who reported lower intentions of taking Calculus II at the end of term compared to the beginning of the term are referred to as Switchers.

In this study we examine the characteristics of students who enroll in Calculus I and either persist or switch out of the mainstream calculus sequence, and hence either remain or leave the STEM pipeline, attending specifically to gender. We perform a mixed-effects logistic regression analysis of student change in their intention to take Calculus II by gender while controlling for students’ preparedness for Calculus I, intended career goals, perception of instruction, and institutional environment. (See S3 File for a complete model description.)

To measure preparedness, we use student reported previous calculus experience and standardized math test score (ACT and SAT). Career goals are characterized by students’ reported career aspirations. Students intending to pursue a career in science, technology, or math are grouped together and labeled STM. This label includes students pursuing a career in the life sciences. Although Calculus II is not mandatory in some life sciences degree programs, it often serves to fulfill higher level mathematics degree requirements and is explicitly required by other programs. We consider students pursuing medical professions, non-STEM fields (e.g. business, law, education), and those who are undecided to be STEM-interested as these students indicated they were originally planning to take Calculus II at the beginning of the term, and thus must have been initially open to pursing a degree that required more mathematics (see S2 Table for more detailed information). The STEM-interested students could be considering a STEM field as a second degree or interdisciplinary studies involving STEM, which are witnessing much greater demands in industry than specialized science fields [35].

Student perception of instruction was characterized by aggregate variables Instructor Quality and Student-Centered Practices, ranging from 1-6, based on student reports of sixteen instructional practices and behaviors (see S3 and S4 Tables for detailed information on the derivation of these variables). Instructor Quality characterizes the level of conventional quality teaching, including availability outside of office hours, listening to questions, and encouraging students mathematically. Low values on this scale indicate low perceived instructional quality, and high values correspond to high instructional quality. Student-Centered Practices characterizes the frequency of classroom practices such as whole-class discussion, students giving presentations, and group work. Low values coincide with traditional, instructor-centered instructional practices, and high values correspond to more innovative, student-centered teaching. Since there are many unknown and unmeasured characteristics of an institutional environment that likely contribute to a student’s career decision, we expect dependence among switcher propensities at the same institution. For this reason, we also included institution in the analysis as a random effect.

## Results

### Identifying who is Switching out of Calculus

Using a logistic mixed-effects regression model, we analyzed the association between switcher propensity and gender, controlling for student preparedness, career intentions, instruction, and institution. A total of 4,065 students were classified as Persisters and 803 students were classified as Switchers. Of these students, there were 2,266 students for which we had complete data and of these 17.8% were identified as Switchers.

**Fig 2:**
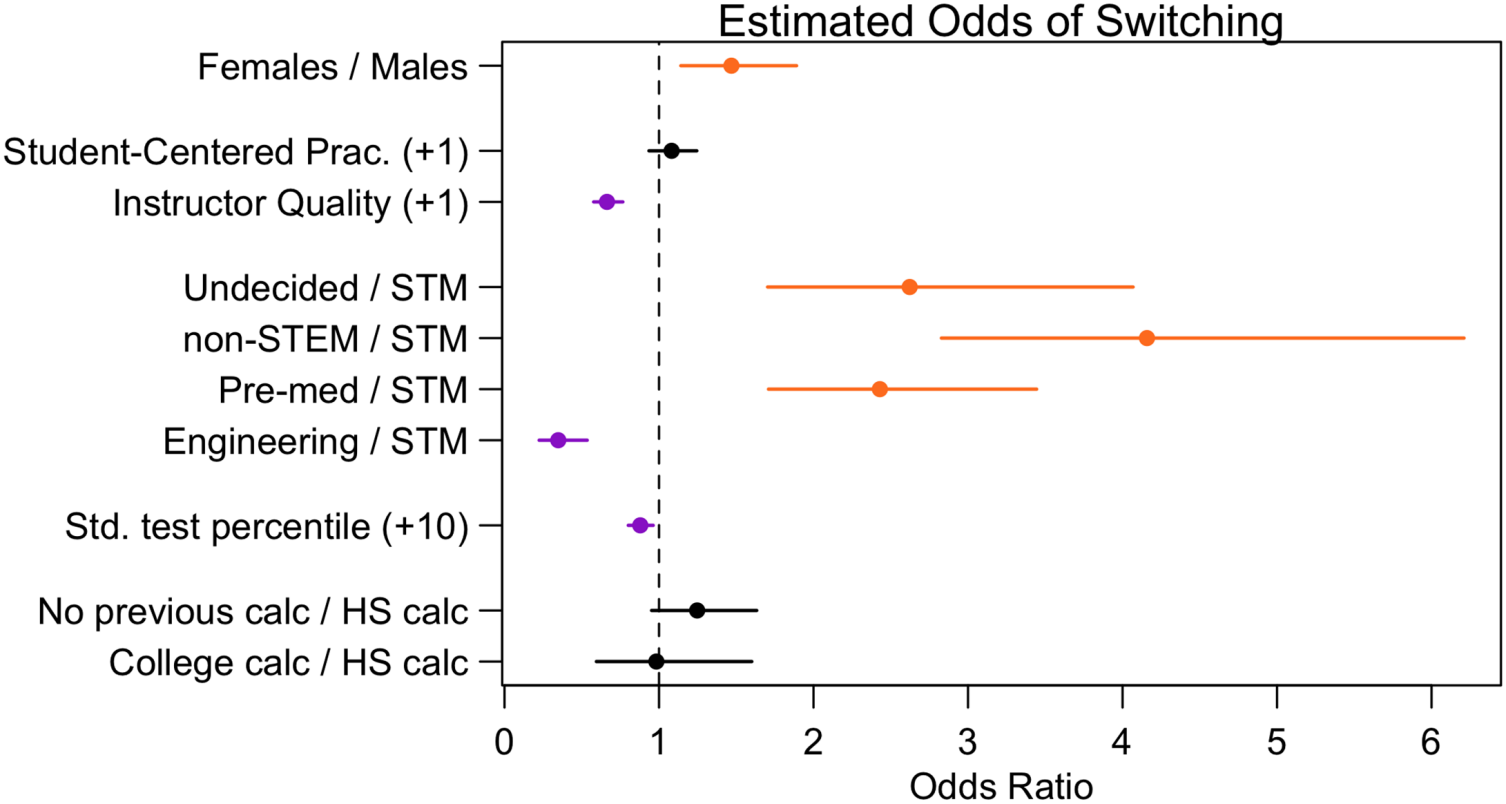
Odds ratios of switching for student attributes. The circle represents the odds ratio estimate and the bars represent the 95% credible interval. The continuous variables noted with (+x) on the left compare a student who reported x-points higher than another student. Labels of the form A / B correspond to the ratio of the odds of switching for a student of type A to the odds of switching for a student of type B. Variables associated with decreased likeliness and increased likeliness of switching are highlighted in purple and orange, respectively. [N = 2266.]

Three of the controlling variables were found to be significant when predicting persistence: standardized test math score, career intentions and Instructor Quality. As shown in Fig 2, higher standardized test scores correspond to an increased likelihood of persisting, as does intending to be an Engineer (compared to a STM field) and higher levels of Instructor Quality. Compared to students pursuing a STM field, students pursuing a medical field, non-STEM field, or are undecided are more likely to switch out of calculus. As previously mentioned, such students represent what may be termed STEM-interested students; these students have not yet chosen a STEM career to pursue but instead, by indicating at the beginning of the term that they intend to take Calculus II, are interested in STEM or in STEM aspects of their fields. By increasing the retention of these students, we effectively add participation in the STEM pipeline while also working to repair the leaks.

Surprisingly, neither previous calculus experience nor Student-Centered Practices are significantly associated with switching propensity (since the credible intervals for the odds ratios contain one). It is worth noting that, at least as of data collection in 2010, across the country we saw little variation in calculus instruction, where the predominant mode of instruction is still very lecture based. Thus, while one may expect that more Student-Centered Instruction would be related to higher persistence, it is possible that there was not enough variation in this variable to see a significant difference related to persistence. Another possibility may be that in the short-term, students have non-positive feelings towards more Student-Centered Instruction because it deviates from their past educational experience and from their expectations for a university class. In a large-scale study it was found that student-centered instruction had little effect on student’s immediate success. However, there was a much larger effect on students’ mathematical dispositions that, in turn, can affect students’ long-term STEM persistence [36].

**Fig 3:**
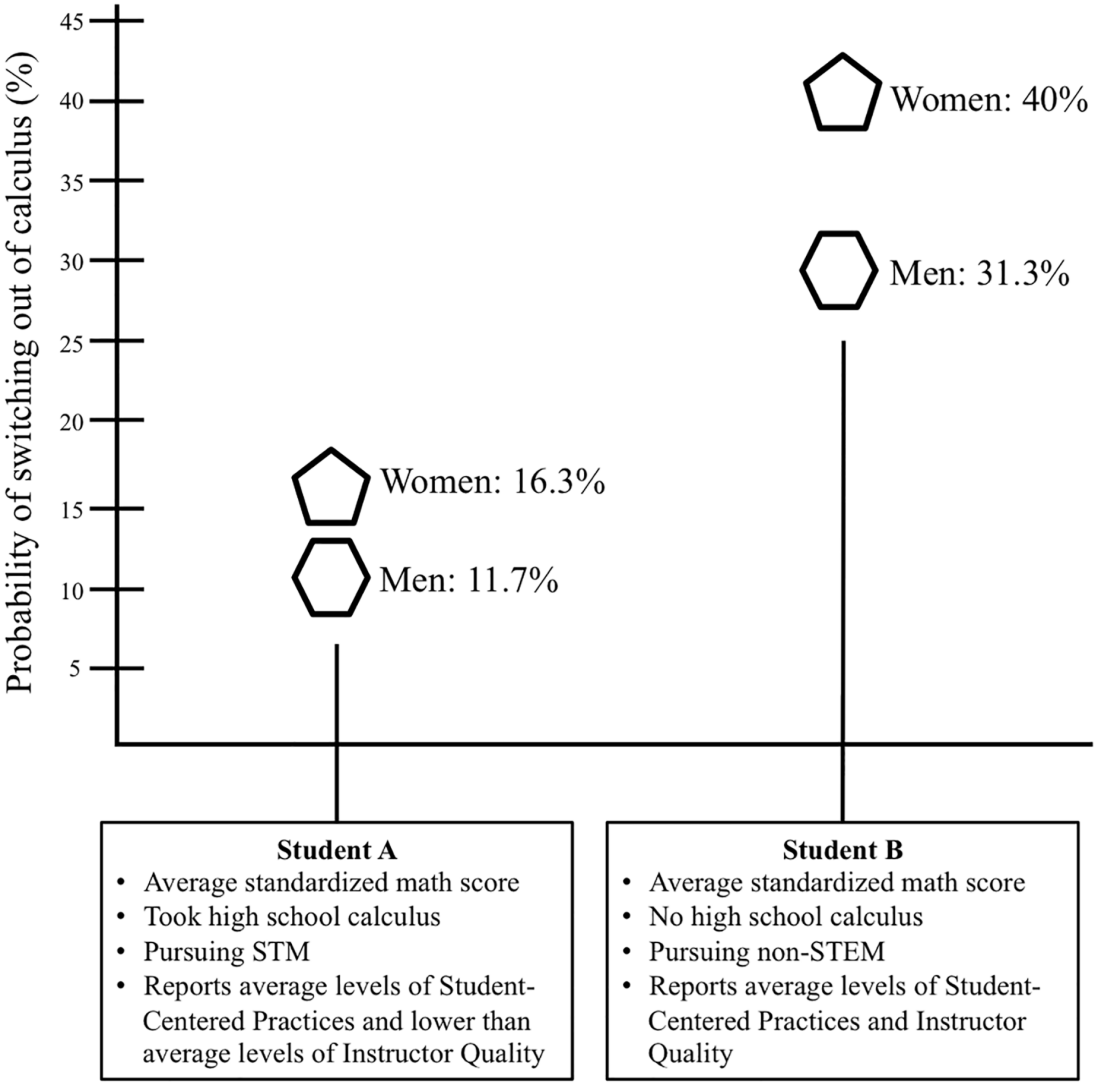
Comparison of probability of switching for two hypothetical students. The hexagon symbol is used to depict a male student and the pentagon symbol is used to depict a female student.

Even after controlling for student preparedness, career intentions, and instruction, gender is significantly related to persistence. Specifically, a female student’s odds of switching are approximately 1.5 times that of a comparable male student of the same preparedness, career goals, and reports of instruction (95% CI: 1.14-1.89) (see S9 Table for more detail). To understand what this means practically, consider two hypothetical students (shown in Fig 3), one STEM-intending and one STEM-interested: Student A earned an average standardized math score, took high school calculus, is pursuing STM, and reports average levels of Student-Centered Practices and lower than average levels of Instructor Quality. If student A is a man, he has an 11.7% probability of switching out of his calculus, whereas if student A is a woman, this probability increases to 16.3%. Student B also earned an average standardized math score, did not take high school calculus, is pursuing a non-STEM career, and reports average Instructor Quality and Student-Centered Practices. If student B is male, he has a 31.3% probability of switching out of his calculus, however if instead, student B is female, this probability increases to 40.0%. These results show that Calculus I is a critical “leak” in the STEM pipeline, especially for women.

### Examining Students’ Reasons for Leaving Calculus

We now consider the question of why. On the end of term survey, students who did not intend to take Calculus II were given a list of potential reasons and were asked to select all that resonated with them. In Table 2, we report statistics on the reasons Switchers gave for not persisting in calculus at the end of Calculus I. We summarize the results for all students that were classified as Switchers and provided gender and STEM intention information. These students represent roughly two-fifths of all Switchers in our study.

**Table 2:** Switchers’ reasons for not intending to take Calculus II. ⋆ indicates gender differences that are statistically significant at the 0.10 level based on Fisher’s exact test. The corresponding *p*-values for STEM-intending and STEM-interested students are 0.026 and 0.051, respectively.[N = 329.]

	STEM-Intending		STEM-Interested	
Reason for not intending to take Calc. II	Men	Women	Men	Women
	(37)	(48)	(86)	(158)
I changed my major and now do not need to take Calculus II	70%	65%	33%	32%
To do well in Calculus II, I would need to spend more time and effort than I can afford	41%	35%	38%	37%
My experience in Calculus I made me decide not to take Calculus II	32%	38%	42%	45%
I have too many other courses I need to complete	27%	25%	50%	50%
I do not believe I understand the ideas of Calculus I well enough to take Calculus II⋆	14%	35%	20%	32%
My grade in Calculus I was not good enough for me to continue to Calculus II	16%	19%	15%	15%

Examining the differences in the reasons selected by STEM-intending versus STEM-interested students provides understanding into why the switching rates among STEM-interested students are much higher compared to STEM-intending students. Among STEM-intending students, the most often selected reason was a change in major. Among STEM-interested students, the most often selected reason was having too many other courses to take. This highlights the priorities of these two groups. STEM-interested students view taking more calculus as secondary to their primary career intention. There may simply not be room in their education plan to pursue their STEM-interests without taking too many courses in one term or staying in school longer, and thus increasing the financial commitment. One approach to increasing participation in the STEM pipeline would be to develop strategies to attract STEM-interested students to take more STEM courses without overburdening their course load or financial commitments. Since a larger majority of STEM-interested students are women, compared to STEM-intending students (see S5 Table), increased retention of STEM-interested students across the board would lead to an increase in women participation in STEM. While not the focus on this paper, this finding is worthy of further examination.

The proportions of students who cited each reason were comparable across men and women, except for one: “I do not believe I understand the ideas of Calculus I well enough to take Calculus II.” Among STEM-intending students, 35% of women reported this as a reason while only 14% of men acknowledged it (*p* = 0.026). Among STEM-interested students, 32% of women reported this as a reason compared to only 20% of men (*p* = 0.051). Thus, women Switchers are citing a lack of understanding of the material in Calculus I as a reason for not continuing their studies significantly more often than men.

In this study, we do not have the data to examine students’ actual abilities. However, previous research suggests that this perceived lack of understanding among women is not because women do not actually understand the material as well as men; on the contrary, a meta-analysis of gender differences in mathematics found no differences in ability [37] and a study specifically looking at gender differences in Calculus I found that women outperform men [38]. The meta-analysis involved all studies comparing mathematical performance, achievement, or ability in algebra, calculus, or geometry by gender from 1990 to 2007, and concludes that gender is not a strong predictor of mathematics performance. In the Calculus I only study, the researchers found that during the 2014 spring semester at the University of Oman, female students outperformed male students in Calculus I. Certainly the second study does not carry the weight of the meta-analysis, but it does provide evidence to counter the common belief that men outperform women in calculus ability.

The gender differences we identified in persistence and reasons for switching are disconcerting as they suggest that perception of one’s ability plays a role in women’s decisions to stop taking calculus but not as much for men, though previous research does not support an actual difference in women’s mathematical ability.

### Investigating Confidence as a Source for Gender Disparities in Switching

It is well documented that confidence and identity play a significant role in one’s success and STEM-intentions [12, 39], and that men and women have different levels of confidence in their mathematical ability [40, 41]. This begs the question of whether calculus is weeding out students based on actual capability or a lack of confidence in their mathematical capability.

**Fig 4:**
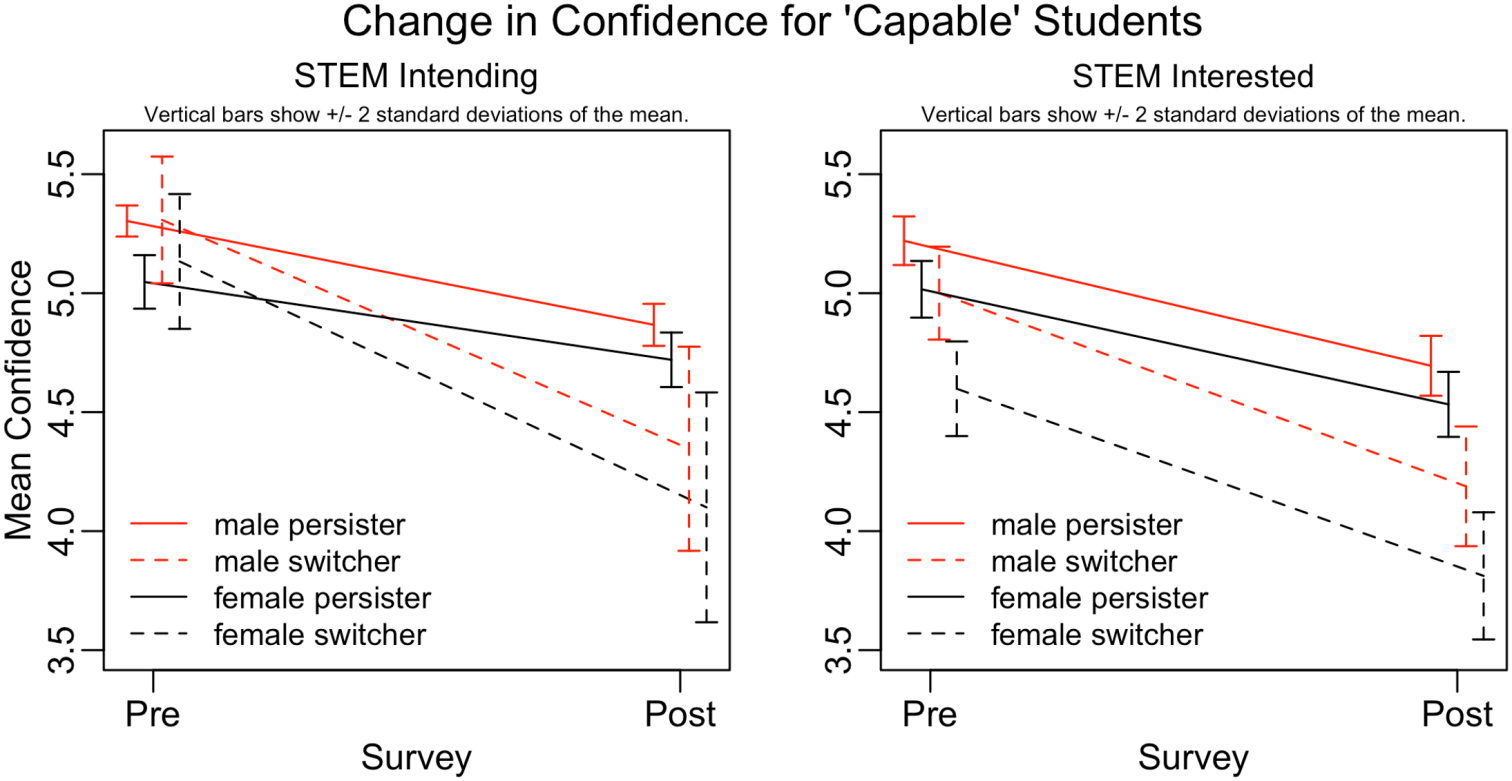
Change in student mathematical confidence at the beginning of the Calculus I semester (pre-survey) and at the end of the semester (post-survey) separated by career intentions, gender, and persistence status. [N = 1524].

To explore this question, we compare the change in student reported mathematical confidence among mathematically-capable students grouped by gender and persistence. We operationalize mathematically-capable as those students with standardized math scores at or above the national 85th percentile. Fig 4 shows that all mathematically-capable students lose mathematical confidence over the course of Calculus I. Switchers experience a greater decrease in confidence than Persisters, and women start at a lower confidence and therefore end at a lower confidence, while experiencing a similar decrease as men. This work points to female students’ mathematical confidence entering college as a major contributing factor to women’s participation in the STEM workforce, and thus more work is needed to understand the factors (such as classroom environment, home environment, extra curricular involvement, etc.,) that help to shape students’ perceptions of their own success before they enter college. Such work is outside the scope of the current study, but our work indicates that significant efforts should be aimed at targeting such questions.

## Discussion

As with any major study, there are limitations in this study related to both the nature of the questions examined and the data set used to answer these questions. We focus on changes in students’ decisions to take Calculus II over the course of a one semester time window, as this decision ultimately reflects students’ intended majors and career paths. Admittedly, a student’s choice to continue pursuing STEM fields is a multifaceted and complex decision involving many more variables than considered here, such as success and enjoyment in other courses taken concurrently with Calculus I, the economy, and extracurricular experiences. This makes it impossible to conclusively identify the role of Calculus I in students’ career decisions without more detailed information. When discussing gender discrepancies in mathematical confidence, we rely on previous research to assert no significant differences exist in mathematical ability between men and women and assume these findings also applicable to our data set. To validate this assumption, ideally this study would have collected measures of student mathematical ability. Unfortunately while final grades were requested, only a very small percentage of instructors submitted them. However, even if such information were available, the variability in grades across institutions (even instructors at the same institution) makes grades unreliable as a measure of ability [42].

**Fig 5:**
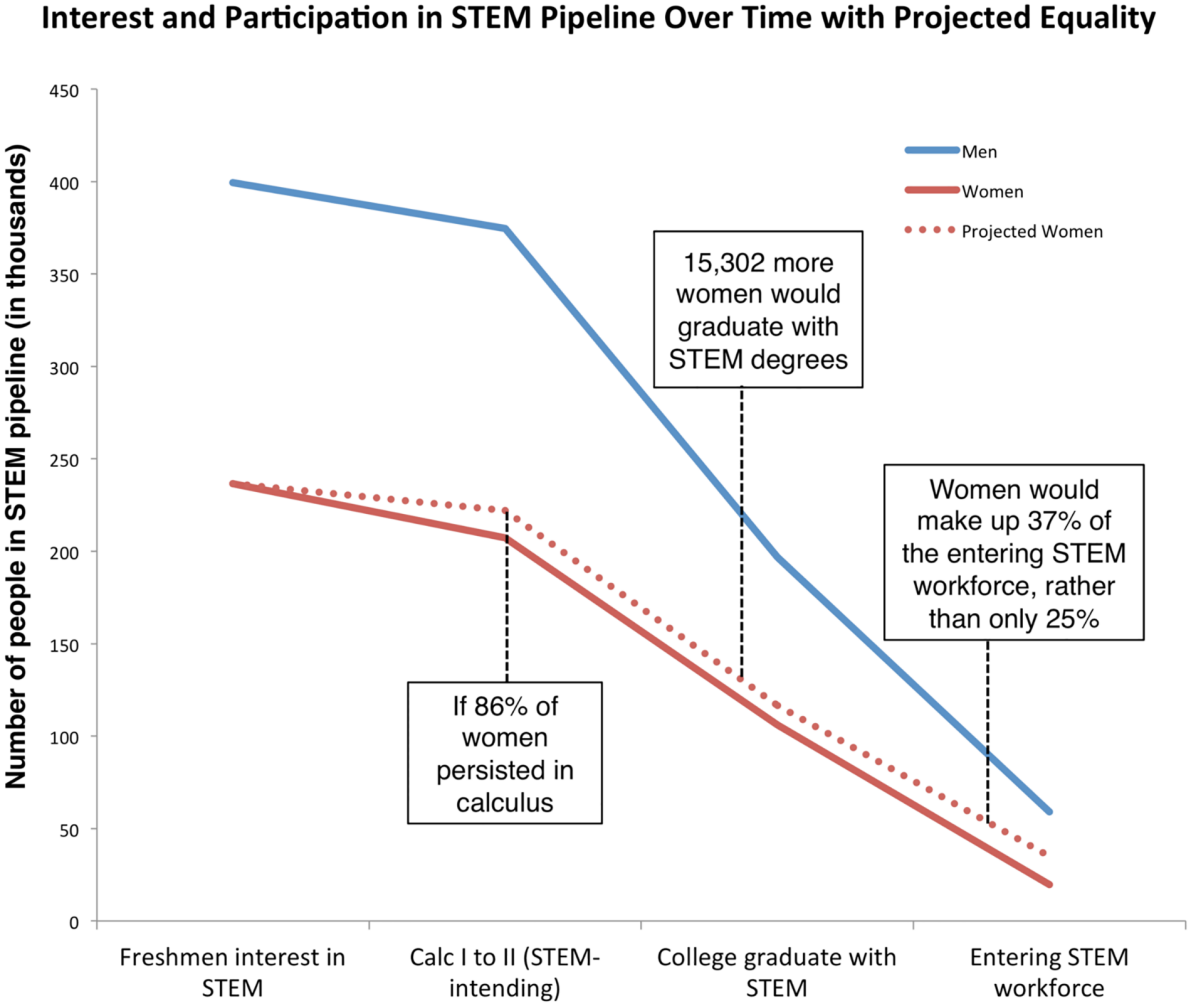
Projected participation of STEM if women and men persisted at equal rates after Calculus I. The dotted line represents the projected participation of women.

Calculus I is an established milestone in the STEM trajectory, and we have shown here that it is contributing significantly to the STEM “gender filter” [43]. What can we do with this information? Our work points to women’s mathematical confidence as a major factor in their decision not to persist in calculus, and therefore STEM. While men and women lose confidence at similar rates during Calculus I, they come into college calculus with different levels of mathematical confidence. Returning to Fig 1, we see young boys and girls expressing similar interests in STEM. Nugent and her colleagues [17] found that one of the most important predictors of STEM persistence was STEM-interest at a young age. Thus, we should expect to see similar participation in STEM in higher education. While there are clear differences in dropout rates between genders from fourth-grade to entering the STEM workforce, substantial gains can be made even if women continue to enter college with lower levels of scientific interest and mathematical confidence. If women persisted in STEM at the same rate as men starting in Calculus I, women would make up as much as 37% of the STEM workforce rather than the current 25%, as shown by the dotted line in Fig 5. Certainly it is preferable to increase girl’s and women’s interest in STEM at all life stages, but this projection indicates that only targeting efforts at college calculus and beyond would increase the number of women entering the STEM workforce by 75%. This would increase the incoming STEM workforce by 20%, and go a long way to meet the needs articulated in the PCAST report [1].

## Supporting Information

S1 Fig Instructor course practices related to (18) “instructor quality” and (19) “student-centered practices”. (PDF)

S2 Fig Trace plots of the *β* coefficients and random effects variance *σ*^2^. The top plot shows the sequence of posterior samples of the regression *β* coefficients and the bottom plot shows that for the random effects variance *σ*^2^.
(TIFF)

S1 File Data File. Data file on which analyses were based. All identifiers have been removed.
(CSV)

S2 File Data File README. File includes information about the survey questions and response codings in data set.
(CSV)

S3 File Supplemental Information File. File including additional information related to the data set, the derivation of Fig 1, data preparation, and data analysis.
(PDF)

S1 Table Switcher coding dictionary outlining student responses to the four questions regarding intention to take Calculus II and their coding as a Switcher or Persister. (PDF)

S2 Table Career choice groupings based on beginning of term survey responses. (PDF)

S3 Table Principal components analysis results for questions related to Instructor Quality. Students were asked to respond to each question on a scale from 1-6, where 1 indicated strongly disagree and 6 indicated strongly agree. The PCA loadings were rescaled to sum to one so that the aggregate variable would range between 1 and 6 like the original questions. *Since the original PCA loading was negative, the last question regarding discouragement to continue in calculus was reverse coded so 1 represents strongly agree and 6 represents strongly disagree.
(PDF)

S4 Table Principal components analysis results for questions related to Student-Centered Practices. Students were asked to respond to each question on a scale from 1-6, where 1 indicated not at all and 6 indicated very often. The PCA loadings were rescaled to sum to one so that the aggregate variable would range between 1 and 6 like the original questions. *Since the original PCA loading was negative, the lecture question was reverse coded so 1 represents very often and 6 represents not at all.
(PDF)

S5 Table Percentage of students that switched out of calculus by career choice and gender. (PDF)

S6 Table Percentage of students that switched out of calculus by previous calculus experience and gender. (PDF)

S7 Table Percentage of students that switched out of calculus by standardized mathematics test percentile and gender. (PDF)

S8 Table Percentage of students that switched out of calculus by aggregate measures of instruction perception and gender. (PDF)

S9 Table Logistic mixed-effects model summary. Odds ratios for switching for categorical variables are presented relative to the reference category noted next to the characteristic (e.g. odds ratio for switching for college calculus is for college calculus compared to high school calculus). The odds ratio for switching for standardized test score compares a student with a test score 10 percentiles higher than another comparable student. Instructor Quality and Student-Centered Practices odds ratios compare perceived instruction for a student rating the course 1 unit higher than another student. Effects with odds ratio credible intervals (CI) that do not contain one are considered to be significant predictors of persistence.
(PDF)
