# Supplementary material for: Women 1.5 Times More Likely to Leave STEM Pipeline after Calculus Compared to Men: Lack of Mathematical Confidence a Potential Culprit
Source: PLoS One. 2016 Jul 13;11(7):e0157447. doi: 10.1371/journal.pone.0157447 (PMC4943602; doi:10.1371/journal.pone.0157447)
Supplement: S1 Fig — (PDF) [file pone.0157447.s001.pdf]

18. My Calculus instructor:

	Strongly Disagree	Disagree	Slightly Disagree	Slightly Agree	Agree	Strongly Agree
asked questions to determine if I understood what was being discussed.	○	○	○	○	○	○
listened carefully to my questions and comments.	○	○	○	○	○	○
discussed applications of calculus.	○	○	○	○	○	○
allowed time for me to understand difficult ideas.	○	○	○	○	○	○
helped me become a better problem solver.	○	○	○	○	○	○
provided explanations that were understandable.	○	○	○	○	○	○
was available to make appointments outside of office hours, if needed.	○	○	○	○	○	○
discouraged me from wanting to continue taking Calculus.	○	○	○	○	○	○

19. During class time, how frequently did your instructor:

	Not at all 1	2	3	4	5	Very often 6
show how to work specific problems?	○	○	○	○	○	○
have students work with one another?	○	○	○	○	○	○
hold a whole-class discussion?	○	○	○	○	○	○
have students give presentations?	○	○	○	○	○	○
have students work individually on problems or tasks?	○	○	○	○	○	○
lecture?	○	○	○	○	○	○
ask questions?	○	○	○	○	○	○
ask students to explain their thinking?	○	○	○	○	○	○
