# Supplementary figures and images for: Women 1.5 Times More Likely to Leave STEM Pipeline after Calculus Compared to Men: Lack of Mathematical Confidence a Potential Culprit
Source: PLoS One. 2016 Jul 13;11(7):e0157447. doi: 10.1371/journal.pone.0157447 (PMC4943602; doi:10.1371/journal.pone.0157447)

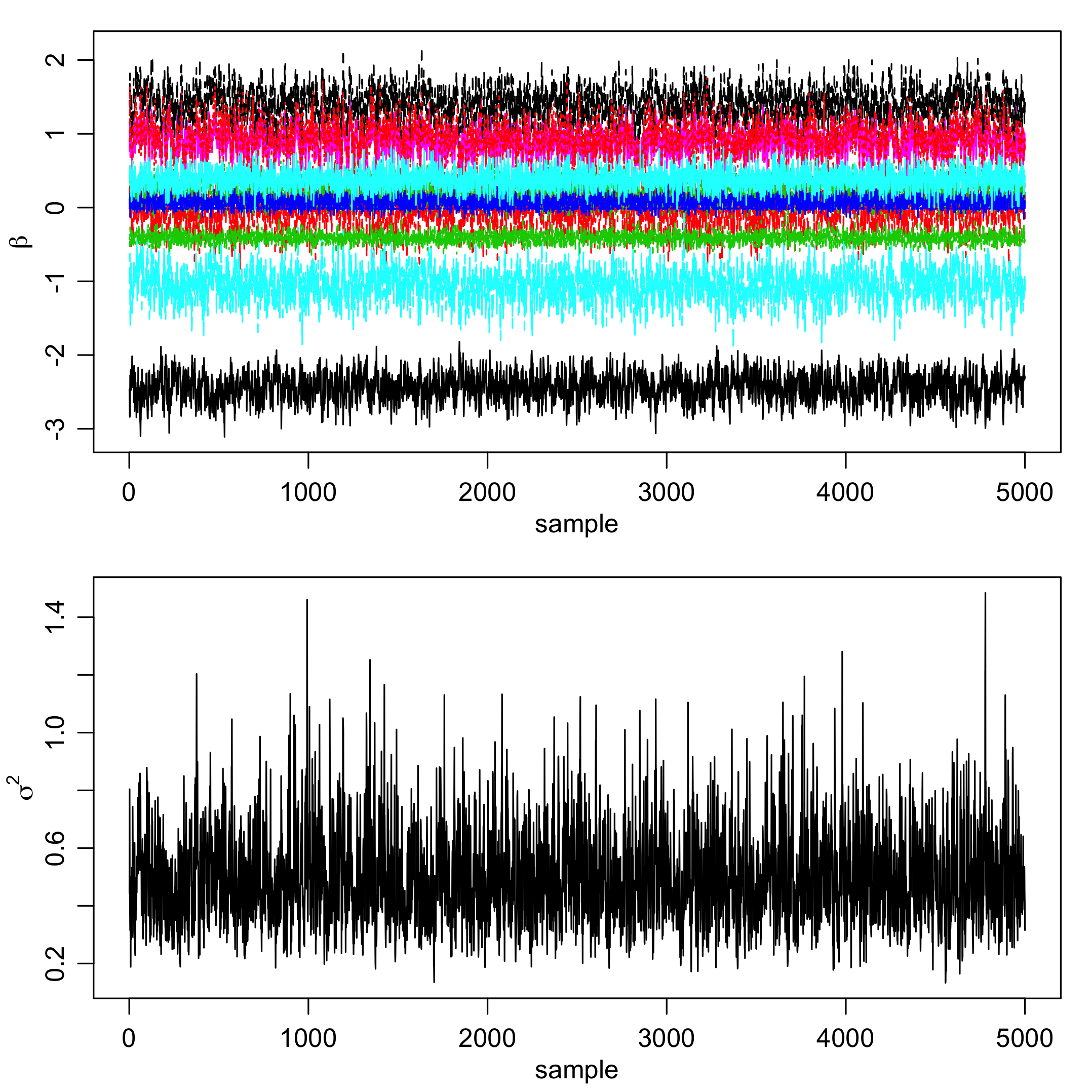

Supplement: S2 Fig — The top plot shows the sequence of posterior samples of the regression β coefficients and the bottom plot shows that for the random effects variance σ2. (TIFF) [file pone.0157447.s002.tiff]
