# Supplementary material for: Women 1.5 Times More Likely to Leave STEM Pipeline after Calculus Compared to Men: Lack of Mathematical Confidence a Potential Culprit
Source: PLoS One. 2016 Jul 13;11(7):e0157447. doi: 10.1371/journal.pone.0157447 (PMC4943602; doi:10.1371/journal.pone.0157447)
Supplement: S3 File — File including additional information related to the data set, the derivation of Fig 1, data preparation, and data analysis. (PDF) [file pone.0157447.s005.pdf]

Women 1.5 Times More Likely to Leave STEM Pipeline After Calculus Compared to Men: Lack of Mathematical Confidence a Potential Culprit

Jess Ellis[1*], Bailey K. Fosdick[2], Chris Rasmussen[3],

1 Department of Mathematics, Colorado State University, Fort Collins, Colorado, USA
2 Department of Statistics, Colorado State University, Fort Collins, Colorado, USA
3 Department of Mathematics and Statistics, San Diego State University, San Diego, California, USA

*** ellis@math.colostate.edu**

Supporting Information

Data Set

The minimal data set for this work is available, titled "Data File", accompanied by "Data File_README" which includes information necessary to use the data.

Fig 1 derivation

The data used in Fig 1 come from a collection of national reports on STEM participation at various milestones, as shown in Table 1 in the manuscript. We began with the number of 4th grade boys and girls in 1999 [1], which was 2,182,000 and 2,025,000 respectively. The National Science Foundation reports that in 2005, 68% of 4th grade boys and 66% of 4th grade girls are interested in science, leaving 1,483,760 boys and 1,336,500 girls [2]. This was the closest projection found for such numbers.

Individuals who completed 4th grade in 1999 would have been entering their senior year in high school in 2007. The census reports that there were 2,149,000 men and 1,998,000 women enrolled in 12th grade in 2007 [3], and the National Center for Education Statistics (NCES) reports 70% of 12th grade men (1,504,300) and 59% of 12th grade women (1,178,820) were interested in science in 2008 (again, the closest projection found) [4].

The census reports that in 2008, there were 2,132,000 men and 2,457,000 women enrolled as first-time freshmen in college [5]. The NCES reports that in 2009, 58% of male college students were enrolled in a 4-year college and 56% of female college students were enrolled in a 4-year college [6]. Thus, 58% of the 2,132,000 undergraduate freshmen men (1,236,560) were at a 4-year college, and 56% of the 2,457,000 undergraduate freshmen women (1,375,920) were at a 4-year college. The American Freshmen report indicates that in 2008, 32.3% of freshmen men and 17.2% of women enrolled in a STEM major [7]. This number includes all biological science, engineering, Physical science, and technical majors. This leads to 399,409 men and 236,658 women planning to study a STEM field in 2012.

The NCES reports that 196,763 men and 106,005 women earned Bachelor's degrees in a STEM field in 2012 [8]. This number includes the biology, computer science,

engineering, mathematics, statistics, and physics degrees. The census reports that only 26% of STEM graduates go on to jobs in the STEM workforce [9], and that only 25% of these jobs are held by women [10]. This leaves a projection of 59,040 men entering the STEM workforce and 19,680 women.

Data preparation

Switcher coding. Students were coded as a Switcher, Persister, or neither based on their responses to four questions. On the beginning of term survey, students were asked if they intended to take Calculus II, with options “yes”, “no” or “I don’t know.” On the end of term survey, students were asked if, at the beginning of the term, they intended to take Calculus II, with options “yes”, “no”, or “I don’t remember” (referred to as “End of term; reflect”). They were also asked if they currently intended to take Calculus II, with options “yes”, “no”, or “I don’t know.” On the follow up survey on year later, students were asked if they had already taken or were currently enrolled in Calculus II, with options “yes” or “no.”

Because not all students answered all surveys, there were multiple ways that we identified students as Switchers, Persisters, or neither. Switchers were identified as any student who provided evidence of decreasing their intentions to take Calculus II. There were 11 unique ways that students could be identified as a Switcher, as shown in S1 Table. Student responses to each of the four questions are filled in as “Y” for “yes”, “N” for “no”, “M” for “I don’t know” or “I don’t remember”, “NA” for not answered, and blank for any option. Thus, in Switcher group 1, students answered “yes” to the beginning of term question, answered anything to the end of term survey questions, and answered “no” a year later. Thus, this group of students entered Calculus I intending to take Calculus II and a year later had not taken Calculus II. Students in Switcher group 8 were initially unsure whether they would take Calculus II, marking “I don’t know”. However, this uncertainty suggests they were at least interested or open to taking more calculus. By the end of the term they said that they did not intend to take Calculus II, and thus they decreased their STEM interest and/or intention.

Students whose responses are not captured in S1 Table were determined to not be initially interested or intending to take Calculus II.

Career choice grouping. On the beginning of term survey students were asked to indicate their intended career choice, choosing from one of 16 options, shown in S2 Table. These intended careers were grouped together into five groups. The first group is comprised of traditional STEM degrees, excluding engineering, the second group. We chose to exclude engineering because there were a disproportionate number of engineering students compared to the other STEM fields. The third group of career intentions is made up of medical and other health professionals. The fourth group is made up of traditionally non-STEM careers, including non-STEM education, social scientists, business, law, humanities, and other non-science related career. The final category is Undecided.

Rather than restrict our analysis to students who indicated that they are intending to pursue a career in STEM, we included all students who indicated that they were at least open to taking more calculus, and thus were either STEM-intending or STEM-interested.

Reports of instruction. On the end of term survey, students were asked two multi-part questions related to instructional practices (see S1 Fig) . Both questions were on a 6-point scale. For the first set of questions, 1 indicated strongly disagree and 6 indicated strongly agree. For the second set of questions, 1 indicated not at all, and 6 indicated very often.

Informed by principal components analyses (PCA), the student reports of the 16 practices were combined to create two aggregate variables called Instructor Quality and Student-Centered Practices. Instructor Quality is intended to represent aspects of instruction relate to good teaching, regardless of instructional approach, while Student-Centered Practices quantifies the instructional approach, focusing on the extent of instruction beyond traditional, lecture-based instruction.

The eight practices from question 18 (see S1 Fig) were averaged based on the PCA loadings to create a new variable called Instructor Quality, with the last prompt (my instructor discouraged me from wanting to continue taking Calculus) reverse coded. Thus, for this new variable a 1 indicates low levels of Instructor Quality and 6 indicates high levels. The loadings from the PCA are shown in S3 Table. In order to create an aggregate variable ranging from 1-6 like the original questions, the PCA loadings were rescaled to sum to one. Rescaling the loadings in this way allows the regression coefficient in the regression analysis to quantifies the change in the propensity for a student to switch out of calculus for a one integer increase in the reported Instructor Quality score. Since the loadings are relatively similar across questions, the new variable can be viewed as the average response of each student to the eight questions.

The eight practices from question 19 (see S1 Fig) were similarly averaged according to their PCA loadings to create a new variable called Student-Centered Practices. Again, for this new variable a 1 indicates low levels of Student-Centered Practices and 6 indicates high levels. S4 Table shows the loadings from the PCA, as well as the rescaled loadings. Since the loading on the lecture question was originally negative, it was recoded so that 1 represents frequent lecture and 6 represents very little lecture. Most of the loadings are similar, except that related to the frequency of instructors showing how to work specific problems and lecturing. Thus, the aggregate variable is effectively an average of the remaining six questions related to instructional approach.

S8 Table shows a summary of student responses on these aggregate variables.

Mathematical preparation. To measure students' mathematical preparation, we use their previous calculus experience and their math standardized test percentile. We group previous experience in calculus into three bins: high school (non-AP, AP AB, or AP BC), college, and none. For standardized test score, students were asked to report their SAT math test score and/or their ACT math test score. Using the college board website and the ACT website [1] reports of percentiles, we converted these scores to an aggregate "standardized math test percentile". For students who reported both, we used the average of their percentiles. See S6 and S7 Tables for summaries of these variables.

Descriptive analysis of switchers

S5-S8 Tables summarize the relationships between student covariates, switcher code, and gender for the 2,266 students for which we had complete data and were included in the logistic mixed-effects model analysis.

Statistical analysis of switchers

A logistic regression model was used to quantify the association between student characteristics and the propensity for students to switch out of the mainstream calculus sequence. Let Y_i be an indicator of whether student i is coded as a switcher (i.e. 1 = Switcher, 0 = Persister). The mixed-effects logistic regression model can be expressed

[1] https://secure-media.collegeboard.org/digitalServices/pdf/sat/sat-percentile-ranks-crit-reading-math-writing-2014.pdf; http://www.actstudent.org/scores/norms1.html

as follows:

$$
\begin{aligned}
P(Y_i = 1) &= p_i; \\
\log\left(\frac{p_i}{1-p_i}\right) &= \beta_0 + \beta_1 \cdot 1[\text{CollegeCalc}_i] + \beta_2 \cdot 1[\text{NoCalc}_i] + \beta_3 \cdot 1[\text{Engineering}_i] \\
&\quad + \beta_4 \cdot 1[\text{Pre-med}_i] + \beta_5 \cdot 1[\text{Non-STEM}_i] + \beta_6 \cdot 1[\text{Undecided}_i] \\
&\quad + \beta_7 \cdot \text{StdTest}_i + \beta_8 \cdot \text{InstructorQuality}_i + \beta_9 \cdot \text{StudentPractices}_i \\
&\quad + \beta_{10} \cdot 1[\text{Female}_i] + \alpha_{\text{Institution}_i}; \\
\alpha_{\text{Institution}_i} &\sim \text{normal}(0, \sigma^2)
\end{aligned}
$$

Student standardized test percentile, previous calculus experience, career goals, course teaching perceptions, and gender are treated as fixed effects and institution is treated as a random effect. The last term in the model expression $\alpha_{\text{Institution}_i}$ represents the institution effect, capturing correlation among students at the same institution, and is assumed to have been generated from a normal distribution.

Career goals, previous calculus experience, gender and institution are categorical variables, while standardized test percentile, Instructor Quality and Student-Centered Practices are continuous. The notation $1[A]$ in the model equation denotes an indicator function which equals one if the argument A is true and zero otherwise. For example, $1[\text{CollegeCalc}_i]$ is equal to one if student i has previous college calculus experience and a zero otherwise. For the categorial variables previous calculus experience, career choice, and gender, one category had to be selected as the baseline category. The categories high school calculus experience, STM career, and male were specified as the baseline categories. The β coefficients corresponding to the categorical variable are interpreted as the change in the odds of switching for each category compared to the respective baseline category. For example, β_1 is the multiplicative change in the log odds of a student switching out of calculus if they have previous college calculus experience compared to previous high school calculus experience. The non-categorical variables, which included standardized test percentile and the aggregate variables instructor quality and student-centered practices, were centered to have mean zero so the baseline student entered Calculus I with an average standardized test percentile and experienced average levels of Instructor Quality and Student Centered Practices.

A Bayesian estimation procedure was employed to estimate the parameters in the mixed-effects logistic regression model. All parameters are assumed to be independent in the prior distribution with the following parametric forms:

$$
\begin{aligned}
\sigma^{-2} &\sim \text{gamma}(1/2, \text{rate} = 1/2), \\
\beta_j &\sim \text{normal}(0, 100) \qquad j = 1, 2, ..., 10.
\end{aligned}
$$

The parameterization and specification of the hyperparameters of the gamma distribution were adopted from that described in [11]. Parameter estimates were obtained using Markov chain Monte Carlo (MCMC) via a Metropolis-Hastings sampling algorithm which samples from the posterior distribution of the parameters given the data (e.g., see [11, 12]). This algorithm iteratively samples the vector of regression coefficients $\{\beta_1, ..., \beta_{10}\}$, vector of random effects $\{\alpha_1, ..., \alpha_K\}$, and random effects variance σ^2 assuming all other parameters are fixed. The Metropolis-Hastings proposal distribution for the regression coefficients is composed of independent normal distributions centered at the current coefficient estimates with a variance of 0.0003. The Metropolis-Hastings proposal for the random effects is also independent normal distributions centered at the current values with a variance of 0.008. These proposal

variances result in acceptance rates of about 25% for the coefficients and 30% for the random effects (see [13, 14] for discussion of optimal acceptance rates). Finally, σ^{-2} is updated from its full conditional (gamma) distribution. R code is provided at the author's webpage.

The MCMC chain was run for five million iterations, with an additional five hundred thousand burn-in iterations. Samples from the posterior were collected every 1000th iteration to reduce dependence in the samples. This resulted in 5,000 samples from the posterior distribution of the parameters given the data, where the effective sample sizes for all parameters was greater than 987. The posterior mean parameter estimates resulting from the MCMC procedure were compared with the maximum likelihood estimates given by the 'glmer' function in the lme4 package in R [15], based on an adaptive Gauss-Hermite quadrature procedure with ten points per axis, and those from GLIMMIX in SAS. The estimates from all three estimation procedures were similar. The MCMC procedure was initialized with the estimates from 'glmer'.

To assess convergence of the Markov chain, we examined trace plots of the parameter samples (see S2 Fig), which suggest reasonable mixing of the chain. Summaries of the posterior distribution of the parameters are given in Fig 2 in the manuscript and S9 Table. The point estimates are the means of the posterior distribution and the 95% credible intervals were created from the 2.5th and 97.5th quantiles. The posterior mean estimate of the intercept (on the logit scale) and 95% credible interval are 0.09 and (0.06, 0.12). Also, the estimate and 95% credible interval for the variance of the institution random effects are 0.49 and (0.25, 0.86).

References

1. U.S. Census Bureau. Single Grade of Enrollment and High School Graduation Status for People 3 Years Old and Over, by Sex, Age (Single Years for 3 to 24 Years), Race, and Hispanic Origin. Retrieved from http://www.census.gov/hhes/school/data/cps/1999/tables.html; 1999.

2. NSF, National Science Foundation, Back to School: Five Myths about Girls and Science. Press Release 07-108. Division of Science Resources Statistics, Arlington, VA; 2007.

3. U.S. Census Bureau. Single Grade of Enrollment and High School Graduation Status for People 3 Years Old and Over, by Sex, Age (Single Years for 3 to 24 Years), Race, and Hispanic Origin. Retrieved from http://www.census.gov/hhes/school/data/cps/2007/tables.html; 2007.

4. Cunningham, B. C., Hoyer, K. M., and Sparks, D. Gender differences in science, technology, engineering, and mathematics (STEM) interest, credits earned, and NAEP performance in the 12th grade (NCES 2015-075). U.S. Department of Education. Washington, DC: National Center for Education Statistics; 2015.

5. U.S. Census Bureau. Single Grade of Enrollment and High School Graduation Status for People 3 Years Old and Over, by Sex, Age (Single Years for 3 to 24 Years), Race, and Hispanic Origin: October 2008. Retrieved from http://www.census.gov/hhes/school/data/cps/2008/tables.html; 2008.

6. National Center for Education Statistics (NCES). Total undergraduate fall enrollment in degree-granting postsecondary institutions, by attendance status, sex of student, and control and level of institution: Selected years, 1970 through 2024. Retrieved from http://nces.ed.gov/programs/digest/d14/tables/dt14_303.70.asp; 2014.

7. Eagan K, Lozano JB, Hurtado S, Case MH. The American freshman: National norms fall 2009. Los Angeles: Higher Education Research Institute, UCLA. 2009.

8. National Center for Education Statistics (NCES). Bachelor's, master's, and doctor's degrees conferred by postsecondary institutions, by sex of student and discipline division: 2012-13. Retrieved from https://nces.ed.gov/programs/digest/d14/tables/dt14_318.30.asp; 2014.

9. U.S. Census Bureau. Census Bureau Reports Majority of STEM College Graduates Do Not Work in STEM Occupations. Retrieved from http://www.census.gov/newsroom/press-releases/2014/cb14-130.html.; 2014.

10. Landivar, L. C. Disparities in STEM employment by sex, race, and Hispanic origin. Educ Rev. 2013; 29(6), 911-922.

11. Hoff PD. A First Course in Bayesian Statistical Methods. Springer Science & Business Media; 2009 Jun 2.

12. Gelman A, Carlin JB, Stern HS, Rubin DB. Bayesian Data Analysis. Boca Raton, FL, USA: Chapman & Hall/CRC; 2014 Sep.

13. Roberts GO, Gelman A, Gilks WR. Weak Convergence and Optimal Scaling of Random Walk Metropolis Algorithms. The Annals of Applied Probability. 1997;7(1):110-20.

14. Roberts GO, Rosenthal JS. Optimal scaling for various Metropolis-Hastings algorithms. Statistical Science. 2001;16(4):351-67.

15. Bates D, Maechler M, Bolker B, Walker, S. Fitting Linear Mixed-Effects Models Using lme4. Journal of Statistical Software. 2015; 67(1):1-48. doi:10.18637/jss.v067.i01.
