# Supplementary material for: Women 1.5 Times More Likely to Leave STEM Pipeline after Calculus Compared to Men: Lack of Mathematical Confidence a Potential Culprit
Source: PLoS One. 2016 Jul 13;11(7):e0157447. doi: 10.1371/journal.pone.0157447 (PMC4943602; doi:10.1371/journal.pone.0157447)
Supplement: S1 Table — (PDF) [file pone.0157447.s006.pdf]

S1 Table. Switcher coding dictionary outlining student responses to the four questions regarding intention to take Calculus II and their coding as a Switcher or Persister.

Switcher Group	Number of Students	Beginning of term	End of term; reflect	End of term	Follow up
1	160	Y			N
2	118	M			N
3	15	NA	Y		N
4	3	NA	M		N
5	38	Y	Y	M	NA
6	123	Y		N	NA
7	17	M	Y	M	NA
8	152	M		N	NA
9	34	NA	Y	M	NA
10	78	NA	Y	N	NA
11	65	NA	M	N	NA

Persister Group	Number of Students	Beginning of term	End of term; reflect	End of term	Follow up
12	586	Y			Y
13	63	M			Y
14	67	NA	Y		Y
15	2	NA	M		Y
16	1543	Y		Y	NA
17	35	Y	M	M	NA
18	5	Y	N	M	NA
19	1	Y	NA	M	NA
20	193	M		Y	NA
21	64	M	M	M	NA
22	22	M	N	YM	NA
23	3	M	NA	M	NA
24	1325	NA	Y	Y	NA
25	53	NA	M	Y	NA
26	103	NA	M	M	NA
