# Supplementary material for: Women 1.5 Times More Likely to Leave STEM Pipeline after Calculus Compared to Men: Lack of Mathematical Confidence a Potential Culprit
Source: PLoS One. 2016 Jul 13;11(7):e0157447. doi: 10.1371/journal.pone.0157447 (PMC4943602; doi:10.1371/journal.pone.0157447)
Supplement: S2 Table — (PDF) [file pone.0157447.s007.pdf]

S2 Table. Career choice groupings based on beginning of term survey responses.

Group	Coding	Original indicated career choice
STM (traditional STEM fields, excluding engineering)	1	Life scientist (e.g. biologist, medical researcher)
	1	Earth/environmental scientist (e.g. geologist)
	1	Physical scientist (e.g. chemist, physicist)
	1	Computer scientist, IT
	1	Mathematician
	1	Science/math teacher
Engineering	2	Engineer
Pre-med	3	Medical professional (e.g. doctor, dentist)
	3	Other health professional (e.g. nurse, technician)
Non-STEM	4	Other teacher
	4	Social scientist (e.g. psychologist, sociologist)
	4	Business administration (e.g. finance, management)
	4	Lawyer
	4	English/language/arts specialist
	4	Other non-science related career
Undecided	5	Undecided
