# Supplementary material for: Women 1.5 Times More Likely to Leave STEM Pipeline after Calculus Compared to Men: Lack of Mathematical Confidence a Potential Culprit
Source: PLoS One. 2016 Jul 13;11(7):e0157447. doi: 10.1371/journal.pone.0157447 (PMC4943602; doi:10.1371/journal.pone.0157447)
Supplement: S3 Table — Students were asked to respond to each question on a scale from 1-6, where 1 indicated strongly disagree and 6 indicated strongly agree. The PCA loadings were rescaled to sum to one so that the aggregate variable would range between 1 and 6 like the original questions. *Since the original PCA loading was negative, the last question regarding discouragement to continue in calculus was reverse coded so 1 represents strongly agree and 6 represents strongly disagree. (PDF) [file pone.0157447.s008.pdf]

S3 Table. Principal components analysis results for questions related to Instructor Quality. Students were asked to respond to each question on a scale from 1-6, where 1 indicated strongly disagree and 6 indicated strongly agree. The PCA loadings were rescaled to sum to one so that the aggregate variable would range between 1 and 6 like the original questions. *Since the original PCA loading was negative, the last question regarding discouragement to continue in calculus was reverse coded so 1 represents strongly agree and 6 represents strongly disagree.

**Question 18 -** My calculus instructor:	PCA loadings	Rescaled PCA loadings
Asked questions to determine if I understood what was being discussed	0.366	0.131
Listened carefully to my questions and comments	0.364	0.131
Discussed applications of calculus	0.305	0.110
Allowed time for me to understand difficult ideas	0.414	0.149
Helped me become a better problem solver	0.410	0.147
Provided explanations that were understandable	0.409	0.147
Was available to make appointments outside of office hours, if needed	0.229	0.082
Discouraged me from wanting to continue taking calculus*	0.286	0.103
