# Supplementary material for: Women 1.5 Times More Likely to Leave STEM Pipeline after Calculus Compared to Men: Lack of Mathematical Confidence a Potential Culprit
Source: PLoS One. 2016 Jul 13;11(7):e0157447. doi: 10.1371/journal.pone.0157447 (PMC4943602; doi:10.1371/journal.pone.0157447)
Supplement: S4 Table — Students were asked to respond to each question on a scale from 1-6, where 1 indicated not at all and 6 indicated very often. The PCA loadings were rescaled to sum to one so that the aggregate variable would range between 1 and 6 like the original questions. *Since the original PCA loading was negative, the lecture question was reverse coded so 1 represents very often and 6 represents not at all. (PDF) [file pone.0157447.s009.pdf]

S4 Table. Principal components analysis results for questions related to Student-Centered Practices. Students were asked to respond to each question on a scale from 1-6, where 1 indicated not at all and 6 indicated very often. The PCA loadings were rescaled to sum to one so that the aggregate variable would range between 1 and 6 like the original questions. *Since the original PCA loading was negative, the lecture question was reverse coded so 1 represents very often and 6 represents not at all.

**Question 19 -** During class time, how frequently did your instructor:	PCA loadings	Rescaled PCA loadings
Show how to work specific problems?	0.095	0.038
Have students work with one another?	0.501	0.201
Hold a whole-class discussion?	0.507	0.203
Have students give presentations?	0.257	0.103
Have students work individually on problems or tasks?	0.373	0.149
Lecture?*	0.051	0.021
Ask questions?	0.252	0.101
Ask students to explain their thinking?	0.460	0.184
