# Supplementary material for: Women 1.5 Times More Likely to Leave STEM Pipeline after Calculus Compared to Men: Lack of Mathematical Confidence a Potential Culprit
Source: PLoS One. 2016 Jul 13;11(7):e0157447. doi: 10.1371/journal.pone.0157447 (PMC4943602; doi:10.1371/journal.pone.0157447)
Supplement: S5 Table — (PDF) [file pone.0157447.s010.pdf]

S5 Table. Percentage of students that switched out of calculus by career choice and gender.

Career choice	Gender	N	Switcher %
STM	Male	263	10.6
	Female	223	16.1
Engineering	Male	539	3.5
	Female	249	6.4
Pre-med	Male	199	21.6
	Female	318	33.3
Non-STEM	Male	136	36.8
	Female	126	38.1
Undecided	Male	99	26.3
	Female	114	28.1
