# Supplementary material for: Women 1.5 Times More Likely to Leave STEM Pipeline after Calculus Compared to Men: Lack of Mathematical Confidence a Potential Culprit
Source: PLoS One. 2016 Jul 13;11(7):e0157447. doi: 10.1371/journal.pone.0157447 (PMC4943602; doi:10.1371/journal.pone.0157447)
Supplement: S6 Table — (PDF) [file pone.0157447.s011.pdf]

S6 Table. Percentage of students that switched out of calculus by previous calculus experience and gender.

Previous Calculus	Gender	N	Switcher %
High School	Male	740	13.0
	Female	699	21.7
College	Male	99	17.2
	Female	62	14.5
None	Male	397	13.4
	Female	269	28.6
