# Supplementary material for: Women 1.5 Times More Likely to Leave STEM Pipeline after Calculus Compared to Men: Lack of Mathematical Confidence a Potential Culprit
Source: PLoS One. 2016 Jul 13;11(7):e0157447. doi: 10.1371/journal.pone.0157447 (PMC4943602; doi:10.1371/journal.pone.0157447)
Supplement: S7 Table — (PDF) [file pone.0157447.s012.pdf]

S7 Table. Percentage of students that switched out of calculus by standardized mathematics test percentile and gender.

Test Percentile	Gender	N	Switcher %	Test Percentile	Gender	N	Switcher %
90-100	Male	711	12.4	40-49	Male	22	18.2
	Female	527	23.0		Female	33	24.2
80-89	Male	247	15.0	30-39	Male	8	12.5
	Female	216	20.8		Female	4	50.0
70-79	Male	164	12.8	20-29	Male	3	0.0
	Female	157	26.1		Female	1	0.0
60-69	Male	50	16.0	10-19	Male	2	50.0
	Female	60	18.3		Female	2	50.0
50-59	Male	26	23.1	0-9	Male	3	0.0
	Female	29	27.6		Female	1	100.0
