# Supplementary material for: Women 1.5 Times More Likely to Leave STEM Pipeline after Calculus Compared to Men: Lack of Mathematical Confidence a Potential Culprit
Source: PLoS One. 2016 Jul 13;11(7):e0157447. doi: 10.1371/journal.pone.0157447 (PMC4943602; doi:10.1371/journal.pone.0157447)
Supplement: S8 Table — (PDF) [file pone.0157447.s013.pdf]

S8 Table. Percentage of students that switched out of calculus by aggregate measures of instruction perception and gender.

Instructor Quality Response	Gender	N	Switcher %	Student-Centered Instruction Response	Gender	N	Switcher %
[5.5, 6]	Male	165	9.1	[5.5, 6]	Male	17	17.6
	Female	152	16.4		Female	21	38.1
[4.5, 5.5)	Male	613	12.2	[4.5, 5.5)	Male	155	12.3
	Female	464	20.3		Female	124	25.0
[3.5, 4.5)	Male	323	13.6	[3.5, 4.5)	Male	363	12.1
	Female	281	25.3		Female	282	22.0
[2.5, 3.5)	Male	95	24.2	[2.5, 3.5)	Male	393	16.3
	Female	89	29.2		Female	296	23.6
[1.5 ,2.5)	Male	33	24.2	[1.5, 2.5)	Male	252	11.1
	Female	37	48.6		Female	257	23.0
[1,1.5)	Male	7	14.3	[1, 1.5)	Male	56	14.3
	Female	7	57.1		Female	50	16.0
