# Supplementary material for: Women 1.5 Times More Likely to Leave STEM Pipeline after Calculus Compared to Men: Lack of Mathematical Confidence a Potential Culprit
Source: PLoS One. 2016 Jul 13;11(7):e0157447. doi: 10.1371/journal.pone.0157447 (PMC4943602; doi:10.1371/journal.pone.0157447)
Supplement: S9 Table — Odds ratios for switching for categorical variables are presented relative to the reference category noted next to the characteristic (e.g. odds ratio for switching for college calculus is for college calculus compared to high school calculus). The odds ratio for switching for standardized test score compares a student with a test score 10 percentiles higher than another comparable student. Instructor Quality and Student-Centered Practices odds ratios compare perceived instruction for a student rating the course 1 unit higher than another student. Effects with odds ratio credible intervals (CI) that do not contain one are considered to be significant predictors of persistence. (PDF) [file pone.0157447.s014.pdf]

S9 Table. Logistic mixed-effects model summary. Odds ratios for switching for categorical variables are presented relative to the reference category noted next to the characteristic (e.g. odds ratio for switching for college calculus is for college calculus compared to high school calculus). The odds ratio for switching for standardized test score compares a student with a test score 10 percentiles higher than another comparable student. Instructor Quality and Student-Centered Practices odds ratios compare perceived instruction for a student rating the course 1 unit higher than another student. Effects with odds ratio credible intervals (CI) that do not contain one are considered to be significant predictors of persistence.

Variable	Odds Ratio	95% CI
Previous calculus: compared to high school calculus		
College calculus	0.98	(0.59, 1.60)
None	1.25	(0.95, 1.63)
Standardized test score:		
Percentile (10 pt increase)	0.88	(0.80, 0.96)
Career choice: compared to STM		
Engineering	0.35	(0.22, 0.54)
Pre-med	2.43	(1.71, 3.44)
Non-STEM	4.16	(2.83, 6.21)
Undecided	2.62	(1.70, 4.07)
Reports of instruction:		
Instructor Quality (1 pt increase)	0.66	(0.58, 0.77)
Student-Centered Practices (1 pt increase)	1.08	(0.94, 1.24)
Gender: compared to men		
Women	1.47	(1.14, 1.89)
